# The Interval Consensus Model: Aggregating Continuous Bounded Interval Responses

**DOI:** 10.1017/psy.2025.10058

**Published:** 2025-11-04

**Authors:** Matthias Kloft, Björn S. Siepe, Daniel W. Heck

**Affiliations:** Department of Psychology, Philipps-Universität Marburg, Germany

**Keywords:** Bayesian modeling, continuous bounded responses, cultural consensus theory, interval responses

## Abstract

Cultural consensus theory (CCT) leverages shared knowledge between individuals to optimally aggregate answers to questions for which the underlying truth is unknown. Existing CCT models have predominantly focused on unidimensional point truths using dichotomous, polytomous, or continuous response formats. However, certain domains, such as risk assessment or interpretation of verbal quantifiers, may require a consensus focused on intervals, capturing a range of relevant values. We introduce the interval consensus model (ICM), a novel extension of CCT designed to estimate consensus intervals from continuous bounded interval responses. We use a Bayesian hierarchical modeling approach to estimate latent consensus intervals. In a simulation study, we show that, under the conditions studied, the ICM performs better than using simple means and medians of the responses. We then apply the model to empirical judgments of verbal quantifiers.

## 1 Introduction

In psychological research, it is common practice to pose questions to respondents for which the correct answer is not known. This may be a forecast of the occurrence of some future event, for example, “that same-sex marriage will be federally recognized by the end of Obama’s term (2017)” (Anders et al., 2014), where the correct answer can in principle be known or will reveal itself eventually. Correct answers are also unavailable in scenarios where the correct answer can change based on the context or the particular group of respondents. For example, one might be interested in judgments of affective valence regarding stimulus words like “accident” (Bradley & Lang, 1999) or in judgments of probabilities assigned to verbal quantifiers like “seldom” or “likely.” Such judgments can often be ambiguous and may systematically vary between groups or individuals, or even within a single individual, depending on the context in which the particular word is used (Karelitz & Budescu, 2004). In such scenarios, it is often of interest to estimate the shared consensus of a certain group by aggregating the given responses.

Cultural consensus theory (CCT) was developed to solve this problem (Batchelder & Romney, 1988; Romney et al., 1986). It is based on the assumption that respondents belong to the same group or subpopulation and share common knowledge about a particular knowledge domain, which is termed the *cultural consensus*. However, respondents may not all have the same level of expertise or background knowledge, and thus, the quality of answers may vary among different respondents. The theory further assumes that weighting responses by expertise will improve the overall accuracy of the aggregated judgments. CCT builds on these assumptions to estimate the cultural consensus by (a) aggregating all responses and (b) weighting each response by the inferred expertise of the respective respondent. To estimate the expertise of the respondents along with the cultural consensus, it is necessary to collect responses to multiple items in the same knowledge domain for each respondent. This can typically be done in a design in which respondents and items are fully crossed, but also in a non-fully crossed design. The consistency of a respondent’s answers across multiple items, relative to the answer patterns of other respondents, is then used to estimate their expertise in the respective domain. Additionally, the discernibility of each item’s cultural consensus is estimated across respondents and incorporated into the estimation of the cultural consensus.

Different consensus models for various combinations of response formats and modalities of the latent consensus have been proposed. The initial consensus model, the general Condorcet model (Batchelder & Romney, 1988), used dichotomous responses to estimate binary consensus values, for instance, for answers on a true–false general knowledge test. Following this, several model extensions have been proposed. The latent truth model (Batchelder & Anders, 2012) also accommodates dichotomous responses, but assumes that the latent consensus values of interest are continuous and lie between zero and one. For instance, respondents were asked for dichotomous judgments indicating whether a disease is contagious (Batchelder & Anders, 2012). While judgments about the perceived contagiousness of a disease can be assessed in a dichotomous response format, true contagiousness is more accurately represented in terms of probability, that is, by a continuous value between zero and one. The latent consensus values thus have a probabilistic meaning, while the observable responses are discrete, binary values of either zero or one. The continuous response model (Anders et al., 2014) extends this model to the case where responses are no longer dichotomous, but rather given on a continuous bounded response scale between zero and one. The model assumes that consensus values are continuous in a latent, unbounded space and are mapped onto the bounded response scale by a logit link function. One application of this model concerns the forecasting of probabilities of future events, such as a large tsunami hitting the coast of a particular country (Anders et al., 2014). Anders et al. (2014) also incorporated a method for estimating multiple cultural consensuses for qualitatively different groups by combining CCT with latent class analysis. Another extension of the latent truth model, the latent truth rater model (Anders & Batchelder, 2015), maps continuous latent consensus values to categorical responses. An example application could be ratings of the grammatical acceptability of English phrases on a seven-point scale (Anders & Batchelder, 2015).

All models described above are unidimensional, as only a single attribute is rated for each item. However, consensus models can also be applied to multidimensional ratings. Mayer & Heck (2023) proposed a model for two-dimensional estimates of geographical locations on maps, where respondents had to estimate the location of cities such as London. In this case, both responses and latent consensus values refer to longitude and latitude and are thus continuous, two-dimensional vectors. In this specific example, the model assumes unbounded coordinates while actual locations are bounded due to geographic constraints such as oceans.

All of the above models assume a single (uni- or two-dimensional) point as the latent, unknown consensus for each item. However, in some domains, a point consensus is too constraining and a range or interval of values may be more appropriate to represent a group’s consensus. One example is the judgment of risks, for example, in cyber-security (Ellerby et al., 2020). When organizations want to determine the attack risk regarding their cyber–physical systems, one way to do this is to have experts estimate these risks for specific system components. The overall estimated risk for a component depends on both the variability of judgments among experts as well as the subjective uncertainty within each expert. While the uncertainty between experts can already be inferred from point judgments, an interval response format provides the opportunity to also incorporate the within-expert uncertainty of a particular risk judgment. In this case, an interval judgment can be conceptualized as an interval of risk estimates ranging from the best-case scenario to the worst-case scenario, that is, a lower and an upper bound of the attack risk of a particular system component. Since every value in such an interval is already a probability, the interval is a range of *plausible* risks. The consensus on plausible risks shared by experts can be of interest to stakeholders, and therefore, plausible risks should be assessed (Ellerby et al., 2020).

Another example concerns verbal quantifiers like “difficult” (Navarro et al., 2016) or “likely” (Karelitz & Budescu, 2004), which might be used to indicate how frequently or with which probability particular events, such as extreme heat waves, are happening (Harris et al., 2017). The use of such quantifiers is ambiguous, since there is no clear-cut convention in terms of numerical probabilities that should be assigned to particular quantifiers (except for words like “always” or “never”). An interval consensus could represent a range of permissible probabilities that a particular word stands for in its pragmatic use.

**Figure 1:**
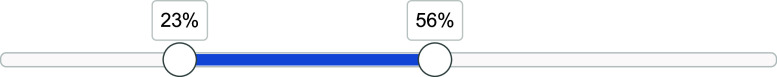
Dual range slider (DRS). *Note*: Screenshot of the *noUiSlider* JavaScript range slider (Gersen, 2024) used in the empirical study (see Section 4). The scale ranges from 0% to 100%.

Interval response formats, such as the dual range slider (DRS) shown in Figure 1, may be a suitable solution for these types of applications. Two sliders allow respondents to judge the lower and upper bounds of a range of values. Ellerby et al. (2022) found that respondents could adequately indicate the variability of different stimuli with an interval response format. In a multi-trait multi-method study, Kloft et al. (2024) found good test–retest reliability of personality scores concerning interval location (reflecting differences in traits between individuals) and interval width (reflecting variability of states within an individual). However, the factor scores for interval widths did not show discriminant validity for the two personality scales used (Extraversion and Conscientiousness). This finding was replicated in another study by Kloft & Heck (2024) in which the DRS response format was applied to different task domains, such as personality adjectives, forecasting of votes, estimation of visual stimuli, estimation of health risks, and judgments of verbal quantifiers. The authors analyzed participants’ interval-width responses in an exploratory factor analysis. Replicating previous results, the discriminant validity of interval widths was low for the two personality scales, as indicated by a common factor for the respective items. However, interval-width responses for the other tasks roughly followed a simple structure with the items of each task loading on a separate factor, suggesting that respondents are sensitive to the requirements of a particular task. Overall, these findings indicate that interval responses are suitable for estimation tasks in which some objectively quantifiable probability or frequency has to be rated. Although various methods for the aggregation of interval ratings have been proposed (Gaba et al., 2017; Lyon et al., 2015; Park & Budescu, 2015), a consensus model, which infers the latent expertise of participants, has not yet been developed for this type of response format. As a remedy, the present article aims to develop a consensus model that can be used to estimate weighted consensus intervals based on ratings collected via continuous bounded interval response formats like the DRS.

We focus on the case where the latent consensus is an interval itself. As discussed by Batchelder & Anders (2012) for unidimensional, dichotomous responses, different kinds of latent consensuses can be mapped onto the same response format used to collect observable ratings. In the case of dichotomous responses, the latent consensus can either be binary, that is, true or false, or continuous, that is, a probability between zero and one of being true or false. Similarly, in the case of collecting interval responses with the DRS response format on a scale from zero to one, the latent consensus can be a single point in 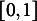 such as the consensus probability of an event happening. However, the latent consensus can also be a consensus interval in 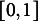 if a range of values is permissible. For instance, in the example of verbal quantifiers, the word “often” could be associated with a consensus interval of 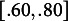 . Which type of latent consensus is more appropriate depends on the substantive application and the psychological constructs of interest (see also Kloft & Heck, 2024, for a discussion of relevant domains and psychological constructs). Regarding models with a point consensus, interval responses are assumed to reflect respondents’ *uncertainty* around their best guess for the unknown value. Regarding models with a latent interval-valued consensus, interval responses are assumed to represent participants’ judgments of the *plausibility* of a range of values (e.g., the consensus range of appropriate probabilities in the example of verbal quantifiers). Also, in the example of judgments of risks, the *plausible range* of a particular risk might be of interest. If we aim at inferring experts’ consensus on the range of plausible risks for a particular event, the desired consensus is an interval.

To facilitate the estimation of consensus intervals, we developed the interval consensus model (ICM), which combines and extends three previous contributions to the literature. The core of the model is the unidimensional consensus model by Anders et al. (2014), which uses a logit-normal distribution to model continuous bounded responses in 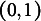 . We extend this model to two dimensions via a bivariate normal distribution, as previously implemented for unbounded responses by Mayer & Heck (2023). Moreover, we use the isometric log-ratio (ILR) transformation function (Smithson & Broomell, 2024) as an appropriate link function that connects the bivariate-normal model to the observed, bounded interval responses.

We explain the mathematical details of the ICM along with a Bayesian estimation method in Section 2 and present a simulation study for the computational evaluation of the model in Section 3. Next, we apply the model in a reanalysis of judgments of verbal quantifiers collected by Kloft & Heck (2024) in Section 4. Lastly, we discuss implications, limitations, and directions for future research in Section 5.

We have implemented the methods presented in this article in the R-package intervalpsych (Kloft & Siepe, 2025). It features functions for data transformation, model fitting, and visualization, as well as the dataset containing judgments of verbal quantifiers.

## 2 Theory

### 2.1 The interval consensus model

In this section, we will introduce the notation for the data and the parameters. Appendix 5 provides an overview of these definitions, along with short explanations. We assume that interval responses are measured on a response scale from 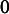 to 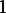 so that the lower and upper interval bounds are given as 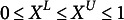 . We first transform the data into a more generalizable compositional form, namely, a simplex with three components that sum to one: 
(1)

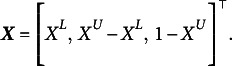

Since any of the three components in 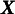 can be zero, we add a padding constant *c* to all components to ensure that we can later apply a log-ratio transformation. After adding the constant, the compositional form is restored by dividing each element of the vector by the sum of all its elements: 
(2)

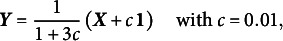

where 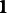 is a vector of three ones. Other methods have been proposed to remove zero components, some of which have properties that are more optimal for compositional analysis, like the preservation of the original ratios of non-zero components for a particular interval response (Martín-Fernández et al., 2003). However, the rescaling method used here has the advantage of preserving the original ratios of non-zero components across *all* responses, which is important for estimating consensus values across items and participants. The rescaling essentially creates a hypothetical response scale for which the extreme values determining the scale’s minimum and maximum cannot occur in the data. The particular choice of 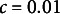 is arbitrary. We conducted a sensitivity analysis (see the materials in the OSF repository), which indicated that this value is a sensible choice. The results in our empirical example (see Section 4) did not change substantially when choosing slightly different values. If none of the components is zero for all responses, we can skip this step in the analysis.

Next, we need to convert interval responses into a format better suited for our modeling framework, which assumes a bivariate normal distribution. For this purpose, we apply a specific version of the ILR transformation function to 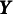 . This link function is tailored to the compositional form of interval responses (Smithson & Broomell, 2024): 
(3)

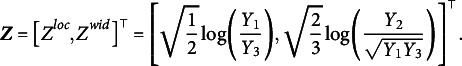

The transformation yields a vector 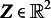 with two elements, 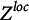 and 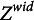 , which correspond to the unbounded interval location and width, respectively. The unbounded interval location 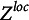 compares the size of the left component 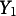 , defined by the left response scale limit and the lower bound of the response interval, against the size of the right component 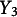 , defined by the upper bound of the response interval and the right response scale limit. The unbounded interval width 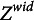 compares the middle component 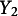 , that is, the observed interval width, to the geometric mean of the left and right components 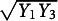 .

The geometric mean in the denominator is used to scale the interval width relative to the interval location in the unbounded space. Therefore, a response interval of a particular width will be transformed into an unbounded interval of a greater width if the interval location is closer to the lower or upper limit of the response scale, compared to being near its center. For example, the response interval 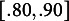 has a mean of the interval bounds of 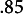 and a width of 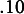 on the bounded scale, which corresponds to a transformed location of 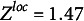 and a transformed width of 
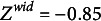
 . Placing an interval with the same observed width near the center of the bounded scale—for example, the interval 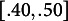 with a mean of interval bounds of 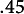 —will yield a considerably smaller transformed width 
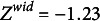
 ( 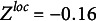 ). This scaling of the transformed, unbounded width, conditional on the interval’s proximity to the response scale limits, accounts for the boundedness of the response scale. To illustrate this, consider a respondent who wants to move the interval location toward one of the response scale limits. Eventually, one of the interval bounds will touch the corresponding response scale limit and it becomes necessary to lower the interval width to move the interval location even closer to the respective response scale limit. The transformation counteracts this effect of the bounded response scale. This is a pragmatic solution that does not necessarily reflect a hypothesized true mapping of a latent response to an observed one. Rather, it is just an assumption similar to the S-shaped item response curves in classical item response models.

**Figure 2:**
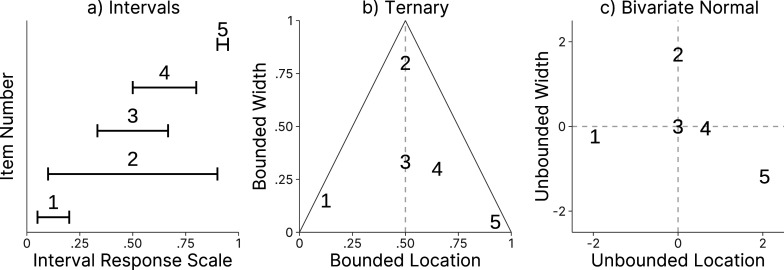
Illustration of the multivariate logit transformation. *Note*: The five observed response intervals are: Interval 1 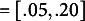 , Interval 2 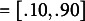 , Interval 3 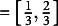 , Interval 4 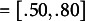 , and Interval 5 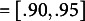 .

Figure 2 illustrates the ILR transformation for five response intervals. Panel (a) shows raw response intervals, Panel (b) represents these intervals in the ternary space, and Panel (c) illustrates their location in the unbounded, transformed space. Interval 3 divides the response scale into a composition of three equally sized components (Panel (a)) and corresponds to the origin of the transformed, unbounded space (Panel (c)). Regarding the location dimension (*x*-axis), the origin in the unbounded space in Panel (c) maps to the center of the bounded response scale in Panel (b). Hence, unbounded location values of zero correspond to response intervals that are centered on the response scale, containing an equal amount of support for values to the left and the right of the scale’s center (e.g., the same proportion of negative and positive values on a bipolar scale). In contrast, the origin of the width dimension (*y*-axis) in the unbounded space does not have such a clear, substantive interpretation. For example, the origin corresponds to a width of one-third when the interval is placed on the center of the response scale. As the interval’s location moves away from the scale’s center, the value zero will correspond to different widths on the bounded response scale. Therefore, the width dimension has slightly different properties than the location dimension, which we will consider below in the parameterization of our model. Interval 2 is also placed on the center of the bounded response scale, but it is much wider, which places it in the center of the *x*-axis and at the upper quarter of the *y*-axis of Panels 2(b) and (c). The other three intervals illustrate how shifts to the left (Interval 1) or to the right (Intervals 4 and 5) on the bounded response scale result in transformed values left and right from the center of the *x*-axis in the unbounded space. As these intervals are relatively small, they have negative values on the *y*-axis in the unbounded space.

The specific form of the ILR transformation that we use here is one of many log-ratio functions described in the compositional analysis literature (Greenacre et al., 2021). In some of these applications, the data have a certain natural meaning (e.g., when studying compositions of chemicals), which is not the case for interval responses. Therefore, not all approaches proposed for compositional data analysis are directly applicable in our case. We need a transformation that yields two conceptually independent and interpretable dimensions, corresponding to the location and the width of response intervals. We know of two log-ratios (described by Smithson & Broomell, 2024) that satisfy these requirements and can thus be applied to interval responses. The first option, the ILR, was presented above. The second option is an amalgamation log-ratio (Greenacre et al., 2021). We tested both log-ratios against each other in a preliminary simulation study (see Section 3) and finally chose the ILR as it performed better. Contrary to the amalgamation approach, the ILR takes the extremity of the interval location into account when determining the transformed interval width, as described above. This is favorable especially in applications with a bipolar response scale featuring a neutral point at the center of the scale, such as a scale ranging from negative to positive values. This may also be a probability scale ranging from 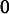 to 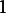 . Here, 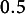 is the neutral point of complete uncertainty, while 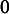 and 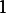 indicate complete certainty about an event not happening or happening, respectively.

Using the ILR transformation as a link function, we can extend the model by Anders et al. (2014) to the two-dimensional case, similar to the model for geographical judgments by Mayer & Heck (2023). We decided to rely on a logit link because it provides more flexibility than the alternative approach of assuming a Dirichlet distribution for the compositional data (see Kloft et al., 2023, for an IRT model using the latter approach). Whereas the Dirichlet approach offers only one common variance parameter for both dimensions, the bivariate logit-normal distribution allows us to assume separate variance parameters for the location and the width dimensions in the unbounded space.

Next, we consider the bivariate, logit-transformed response 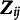 of respondent 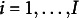 (number of respondents) to item 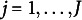 (number of items). We assume the following data-generating mechanism for 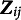 : Respondent *i* makes a latent appraisal, 
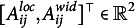
 , for the item *j* based on the latent cultural consensus interval, 
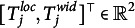
 . This latent appraisal contains some error, which depends on the proficiency of the person, 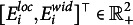 , and on the discernibility of the latent consensus for the particular item, 
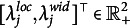
 . Departing from previously developed CCT models (e.g., Anders et al., 2014), we inverted these parameters. Hence, higher values of proficiency and discernibility lead to higher precision of the latent appraisal, and thus, to observed response intervals that are closer to the latent consensus interval. Moreover, we assume an item-specific correlation 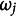 between the errors on the two dimensions (Mayer & Heck, 2023). Assuming a bivariate normal distribution of errors, the appraisal is centered on the latent cultural consensus with an added disturbance governed by person and item characteristics: 
(4)

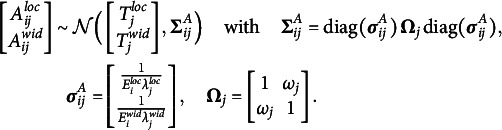



The latent appraisal is further influenced by the respondent’s scaling bias, 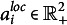 , and shifting biases, 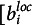 , 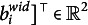 , which yields the final response: 
(5)

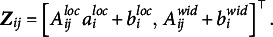

The two shifting biases are directional response biases and add a constant to each dimension of the latent appraisal—or, more technically, to the expected location and width. This corresponds to a respondent’s tendency to systematically under- or over estimate all locations or all widths of the consensus intervals. The scaling bias corresponds to an extremity response bias, which pushes all observed responses of a person away from zero if 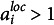 or pulls them toward zero if 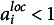 . For the bounded response scale, this means that interval locations are either pushed away from or pulled toward its center. As explained above, the origin of the width dimension, 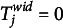 , is not a substantively meaningful anchor, as it depends on the location of a particular interval. It would not be meaningful to let the interval width scale around such an ambiguous, arbitrary value of zero. We thus specify a scaling bias only for the location dimension.

Since the appraisal of the interval location 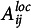 consists of the consensus location plus an error, the scaling bias does not only influence the expected interval location, but also the residual variance, that is, the precision of the latent appraisal. In the full model, it is therefore necessary to ensure that the scaling bias parameter is included not only in the mean but also in the variance of the normal distribution: 
(6)

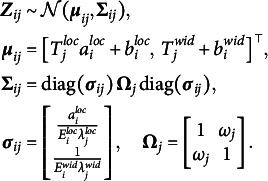

The model can easily be modified by omitting bias parameters that are not relevant for certain applications (see also Section 4.1). Our own workflow involves first fitting the full model and then examining the parameter estimates for any problems. For example, if all respondents have a similar estimate for the location shift bias, 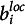 , we remove this parameter from the model.

**Figure 3:**
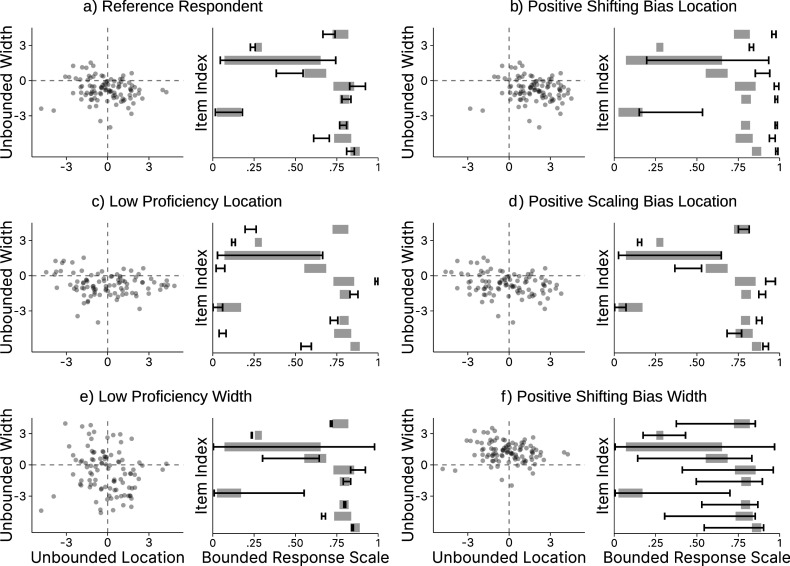
Illustration of how changing the person parameters in the interval consensus model influences the predicted responses. *Note*: The scatter plots in the left-hand subpanels show simulated responses of one respondent to 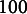 randomly drawn items on the unbounded, bivariate scale. The right-hand subpanels show the corresponding responses (black intervals) for ten selected items on the bounded response scale. The consensus intervals, which are identical across all plots, are shown as gray, shaded bars in the background of the response intervals. We first simulated consensus intervals with 
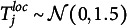
 and 
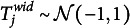
 . Next, we simulated the response intervals in Panel (a) by setting respondent proficiency as well as item discernibility to 1 and assuming no response biases. In the remaining panels, we adopted the hypothetical responses from Panel (a) while manipulating different person parameters (e.g., shifting and scaling biases) to illustrate their effect on response behavior. We lowered the respondent’s proficiencies by factor 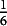 (Panels (c) and (e)), increased the shifting bias by adding a constant of 2 (Panels (b) and (f)), and increased the scaling bias by factor 1.5 (Panel (d)).

Figure 3 shows the isolated influence of each person parameter when all remaining parameters are held constant (see the figure note for more details on the simulation of the shown response patterns). For a person with a low proficiency concerning interval locations ( 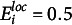 ), Panel 3(c) shows that response intervals move away from the latent consensus interval unsystematically due to increased error variance. Similarly, for a person with a low proficiency concerning interval widths ( 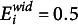 ), Panel 3(e) shows that the widths of response intervals become less similar to the widths of the latent consensus intervals. Inducing a large scaling bias ( 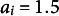 ) for interval locations shifts response intervals away from the center of the scale (Panel 3(d)). A positive shifting bias ( 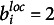 ) for locations, shown in Panel 3(b), moves all response intervals to the right. Similarly, for a positive shifting bias concerning interval widths ( 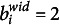 ), Panel 3(f) shows that all response intervals are greatly expanded in width.

It is difficult to interpret the estimate for the latent consensus interval, 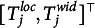 , on the transformed, unbounded scale. To facilitate a substantively meaningful interpretation of this estimate, we convert the unbounded interval back to the original, bounded response scale. First, we transform the two-dimensional logit values to the compositional format via the inverse of the ILR function, and second, we undo the padding initially applied in Equation (2): 
(7)

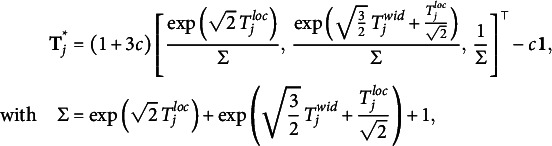

where, again, 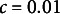 and 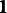 is the vector of ones. If no padding was applied before fitting the model, we set 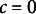 . Third, we compute the actual interval boundaries on the bounded scale from 0 to 1: 
(8)

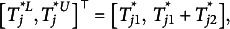

with 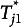 being the first and 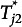 the second component of the simplex 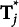 . The interval formed by 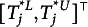 is the estimated consensus for the specific item, which we are ultimately interested in.

### 2.2 Bayesian estimation

We estimate the model in a Bayesian hierarchical modeling framework (Kruschke & Vanpaemel, 2015). An illustration of the prior distributions can be found in the OSF repository and an example comparison of prior and posterior distributions is displayed in Section 4. The main parameters we are interested in are the latent consensus location and width, 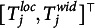 . The priors for these parameters will partly serve to identify the model. To facilitate the specification of these priors, we first specify them on the bounded scale. Then, we transform the values back to the unbounded scale via the ILR function to use them in the model. With this approach, there is no need to define priors on the transformed scale, that is, normal distributions, that align with our assumptions about the implied priors on the bounded scale. From a practical standpoint, we also experienced sampling to be more stable with priors on the bounded instead of the unbounded scale. For the other parameters, which are more flexible due to their hyperpriors, we specify the priors directly on the unbounded scale.

First, based on common applications of interval responses (e.g., in Ellerby et al., 2022; Kloft & Heck, 2024), we assume that consensus intervals with a very large width spanning the entire response scale are highly unlikely. Wide intervals are also not relevant or meaningful in most scenarios, as they would not provide any additional information. Typically, we would exclude items for which we anticipate this to be the case. Therefore, we assign a weakly informative prior to the widths of consensus intervals on the bounded scale: 
(9)

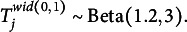

This prior has an expected value of 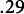 and a mode of 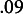 and therefore reflects our beliefs about the marginal width of true intervals more adequately than a uniform prior. However, interval responses of full width (ranging from zero to one) are still possible and not ruled out by our prior choice. Instead, we merely assume that the latent consensus interval itself is unlikely to span the entire response scale. Researchers who want an uninformative uniform prior on the consensus of the interval width may change the prior to 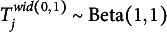 .

Second, conditional on a particular width of a consensus interval, we do not assume that particular locations of the consensus interval are more likely than others. This assumption makes the prior choice more generalizable across different use cases (more informative alternatives are mentioned below). Therefore, we assign an uninformative prior to an auxiliary, multiplicative parameter 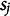 , which is subsequently used to compute the actual interval bounds on the bounded scale: 
(10)

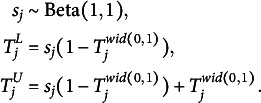

This means that, for a given interval width, we take what is left of the response scale and multiply it by 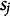 , which results in the lower bound for this particular interval. To arrive at the upper bound, we add the interval width to the lower bound. In the location dimension, we could also choose alternative priors that would be more informative. If theory or prior knowledge suggests that locations in the center of the response scale are more probable, we might choose 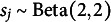 . If we think that locations to the extreme ends of the response scale are more probable, we might choose 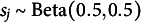 . Such prior knowledge may be informed, for example, by the selected items. For judgments of verbal quantifiers, for example, when only selecting low-probability words like “seldom” or “unlikely,” we can incorporate prior knowledge by giving more weight to consensus locations on the left side of the response scale, for example, 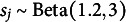 .

Third, we transform the consensus interval from the bounded simplex to the unbounded bivariate scale via the ILR function in Equation (3): 
(11)

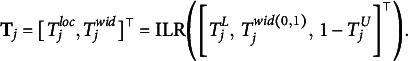

Alternatively, we could have also applied an uninformative prior directly on the simplex via a Dirichlet distribution (an implementation of this prior can also be found in the OSF repository): 
(12)

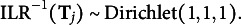



The person proficiency parameters, 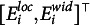 , have weakly informative priors on both the means and the variances (see Table 1, column 1). The priors are specified on the log-scale to ensure positive values. We are also interested in the relationship between a respondent’s proficiency in the location dimension and their proficiency in the width dimension, and therefore assign a bivariate normal prior with correlation parameter 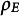 instead of two independent normal priors. Similarly, we assign the same priors to the item discernibilities, 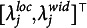 (Table 1, column 2). The only difference is that we fix the mean vector 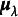 to zero to render the person proficiency parameters identifiable (Anders et al., 2014).

**Table 1:** Default prior distributions for the interval consensus model

Person proficiency 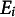	Item discernibility 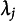
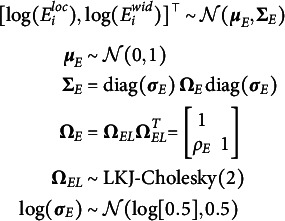	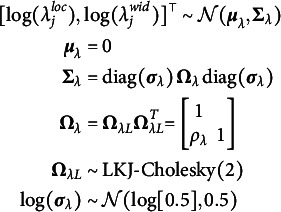

For the remaining person parameters, namely, the scaling and the shifting biases, we also assign weakly informative priors. In doing so, we impose certain restrictions on the means for reasons of identifiability (Anders et al., 2014): 
(13)

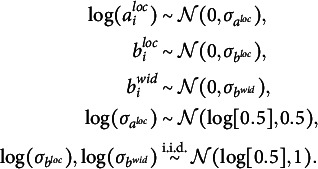

The mean vector of the shifting bias parameters, 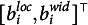 , is fixed to zero to make the model identifiable with respect to the estimated mean of the latent consensus locations and widths. Analogously, the mean vector of the scaling bias parameters, 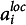 , is fixed to one to render the model identifiable regarding the estimated mean of the proficiency parameters, 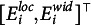 .

Finally, we assign weakly informative priors to the residual correlation between interval location and width via a scaled beta distribution: 
(14)

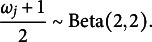



## 3 Simulation study

The simulation study was preregistered at the Open Science Framework (https://osf.io/nd5wg) using the ADEMP preregistration template by Siepe et al. (2024) to specify the Aims, Data-generating mechanisms, Estimands, Methods, and Performance measures. After running the simulation with the pre-registered settings, we found that some conditions resulted in many problematic model fits with divergent transitions of the sampler. We therefore decided to re-work the parameterization and priors of the model for more stable model estimation, and subsequently re-ran the simulation. We indicate deviations from the preregistered settings where applicable. We further provide a list of all deviations and their justification as well as all results of the original simulation study in the OSF repository. The main results did not change, as both the best-performing model per condition and the overall trends of the performance measures remained the same. The simulation study was carried out in the programming environment R Version 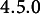 (R Core Team, 2023) on a Linux machine with an Ubuntu 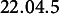 LTS distribution. We provide a Dockerfile to facilitate full reproducibility of our main results. We used the following R packages in their most recent versions at the time of running the simulation: SimDesign (Chalmers & Adkins, 2020) for setting up and conducting the simulation study, cmdstanr (Gabry et al., 2023) as the R interface to Stan (Stan Development Team, 2023), and the posterior (Bürkner et al., 2023) and bayesplot packages (Gabry & Mahr, 2024) for handling and visualizing MCMC output. The specific package version numbers and additional packages used for data wrangling and minor tasks are provided in the OSF repository.

### 3.1 Aims

The simulation study aimed to explore the estimation performance of the ICM concerning bias and mean-squared error of parameter estimates in realistic scenarios of use. The main target estimates were the latent consensus interval location and width, 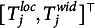 . We also tracked the performance of the other parameters, except for the hyperparameters. We compared the model estimates of the latent consensus intervals for each item against simple means and medians (only means in the pre-registration) of the logit-transformed responses as a simple competitor model (i.e., wisdom of crowds; Surowiecki, 2004). Given that the data were generated from our model, we expected the model estimates to perform better than simple means and medians. If that was not the case, the added complexity of our model may not be worth the effort compared to relying on simpler descriptive aggregation strategies. We further expected that larger numbers of respondents would lead to better performance of item parameters, and, vice versa, that larger numbers of items would lead to better performance of person parameters.

In addition to the main simulation study, we conducted a preliminary simulation study to test the ILR function against an alternative amalgamation log-ratio transformation, which is based on a stick-breaking procedure (see Smithson & Broomell, 2024). We were interested in checking the robustness of the two link functions regarding model misspecification. We generated data with one fixed combination of 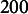 respondents and 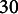 items and only varied the link function used to simulate the data, resulting in two conditions. Each model was fitted to the data using the data-generating link function as well as the non-data-generating link function. We report the full results of this preliminary simulation study in the OSF repository.

### 3.2 Data generation

We randomly generated data from the model described in Section 2.1. We varied the following factors in a fully factorial manner:

- Number of respondents: 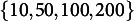 ;
- Number of items: 
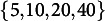
 .

This yielded 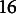 conditions. The numbers of respondents and items were selected to cover a range of practically relevant applications. There may be scenarios with only a few items and few expert raters, for instance, when a company has ten expert employees judging the risk of a security breach for five software components. In other scenarios, large numbers of raters and items might be available, for instance, in a forecasting challenge.

In all conditions, the true, data-generating parameters were randomly drawn for each repetition. We used the model described in Section 2.1 as the data-generating mechanism for each interval response 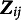 of respondent *i* to item *j* on the unbounded scale. To obtain manifest interval responses in the bounded simplex space, we first transformed the unbounded interval response 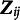 using the inverse ILR function (Smithson & Broomell, 2024). In the model estimation step, the data were then transformed back to the unbounded space using the ILR function (Equation (7) with *c* = 0, see also Smithson & Broomell, 2024). This back-and-forth transformation is a redundant step for fitting the model in our main simulation study, where the same transformation was used for data generation and model estimation. However, for our preliminary simulation study investigating the performance of different link functions, this is a crucial step required to cross-fit a model version with one link function to the data generated with the respective other link function.

Table 2 lists all hyperparameter values used for generating person- and item-specific model parameters. The preregistration protocol contains a detailed justification of these values (see also the corresponding script in the OSF repository). Overall, we aimed to generate plausible distributions of manifest response intervals. We derived the hyperparameters from theoretical response intervals representing typical or extreme responses. For the true mean of consensus interval location and width, we used the values resulting from the logit-transformed interval 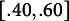 . For the standard deviation of the true consensus interval location, we used the interval 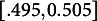 transformed to the bivariate space. We declared the resulting unbounded value as the point that is four standard deviations away from the unbounded mean location, that is, an extreme value in the unbounded space. We further calculated the standard deviation for the unbounded true consensus location by dividing the difference between this extreme value and the mean of the true consensus location by four. Analogously, for the standard deviation of the true consensus width, we used the interval 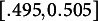 . We simulated the true consensus location and width parameters from normal distributions since all parameters were defined on the unbounded scale. The hyperparameters for the bias parameters were then chosen to yield plausible distributions of the simulated response intervals.

**Table 2:** Values of the hyperparameters used for data generation

Interpretation	Parameter	Distribution / Constant
*Items*		
Consensus location	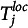	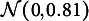
Consensus width	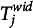	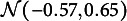
Location discernibility	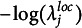	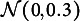
Width discernibility	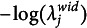	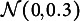
Residual correlation	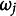	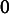
*Respondents*		
Location proficiency	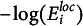	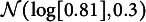
Width proficiency	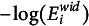	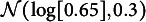
Location scaling bias	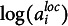	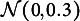
Location shifting bias	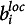	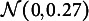
Width shifting bias	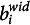	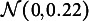

*Note*: For the parameters 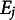 and 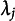 , we defined the distributions on the negative log scale to facilitate an interpretation in terms of the variance instead of the precision.

Due to the large computational demand of our simulation study, we determined the number of repetitions as follows: We aimed for a Monte Carlo standard error (MCSE) of 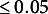 for our primary performance measure (the absolute bias for the latent consensus interval location and width) in all conditions. We deemed 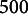 (pre-registration: 1,000) repetitions computationally reasonable. After 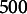 repetitions, we checked the MCSEs in all conditions. If they had not met the above criterion, we would have incrementally added repetitions in steps of 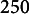 until they did. The MCSEs in all conditions had met the criterion after 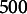 repetitions, with the largest MCSE of the absolute bias being 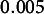 in one condition.

Further details can be found in the preregistration and in the OSF repository, where we illustrate the distributions of parameters and responses as well as the recovery of one set of data-generating parameters.

### 3.3 Method

We estimated the same model for all generated data sets in a Bayesian framework using Stan (Stan Development Team, 2023) in R (R Core Team, 2023) via rstan (Stan Development Team, 2024). For the Bayesian estimation, we used the priors described in Section 2.2, which we did not preregister. The only deviation from the model described above was that we used independent univariate prior distributions instead of a multivariate prior for 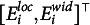 and 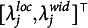 , meaning that we did not estimate the correlations 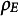 and 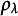 in the simulation. For each repetition, we ran four chains of Stan’s Hamiltonian Monte Carlo sampler (Betancourt, 2018) with 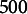 warm-up samples not used for analyses and 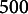 (preregistration: 1,000) samples for the computation of parameter estimates, which yielded 2,000 samples per parameter. Given the results for the convergence diagnostics shown below, we deemed this number sufficient. The adapt_delta parameter was set to 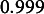 for conditions with a number of total simulated responses 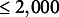 , and to 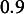 for the conditions with a greater number (preregistration: 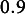 for all conditions). We changed this setting because in our earlier simulations, we had encountered issues with divergent transitions in conditions with low numbers of responses. The range of the initial values of the sampling algorithm for the unbounded parameters was set to 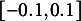 .

### 3.4 Performance measures

Our primary performance measure was the absolute bias of both the latent, unbounded consensus interval location and the width jointly, 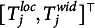 , which we defined as follows: 
(15)

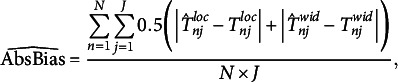

where *J* is the number of items in a specific condition and *N* is the number of repetitions of the simulation. We computed the mean of the (absolute) bias of location and width jointly because we expected that there could be a compensatory effect concerning the accuracy of estimates. We also computed the absolute bias for both dimensions separately for illustration purposes below (see the OSF repository for a plot of the joint biases).

We additionally calculated the mean squared error (MSE) for the bivariate vector 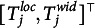 of the latent, unbounded consensus intervals: 
(16)

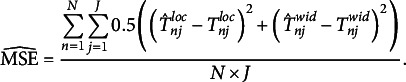

We also calculated the MSE for the location and width individually.

As a measure of parameter recovery, we also computed the average Pearson correlation between the estimated and the true values of the parameters: 
(17)

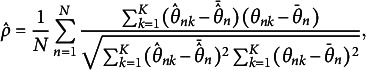

where 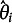 and 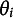 represent the estimated and true values, respectively, 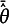 and 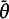 are their respective means, and *K* represents either the number of items or the number of persons, depending on the type of parameter.

We estimated the MCSE of these performance measures via bootstrapping. We further tallied the number of non-converged simulation repetitions.

### 3.5 Results

#### 3.5.1 Preliminary study: Link functions

Our preliminary simulation that compared two alternative link functions showed the superiority of the ILR transformation over the amalgamation log-ratio transformation. Especially in the case of cross-fitting the model to the data generated by the respective other link function, the ILR transformation was more robust to this specific type of model misspecification.

#### 3.5.2 Main study: Recovery of latent consensus intervals

All repetitions of the simulation study finished without error. The average 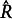 across all repetitions and conditions was 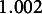 . In 13 of 16 simulation conditions, we observed no divergent transitions. In the “worst” condition with ten respondents and five items, 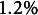 of all models contained at least one divergent transition. The models with divergent transitions in this condition contained on average 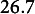 divergent transitions. Overall, these results imply good convergence in almost all repetitions. This indicates that the model can even be estimated in edge cases with a low number of items and respondents, where the performance benefit compared to the aggregation via simple means or medians is particularly large. Additional results on convergence metrics are available in the OSF repository.

**Figure 4:**
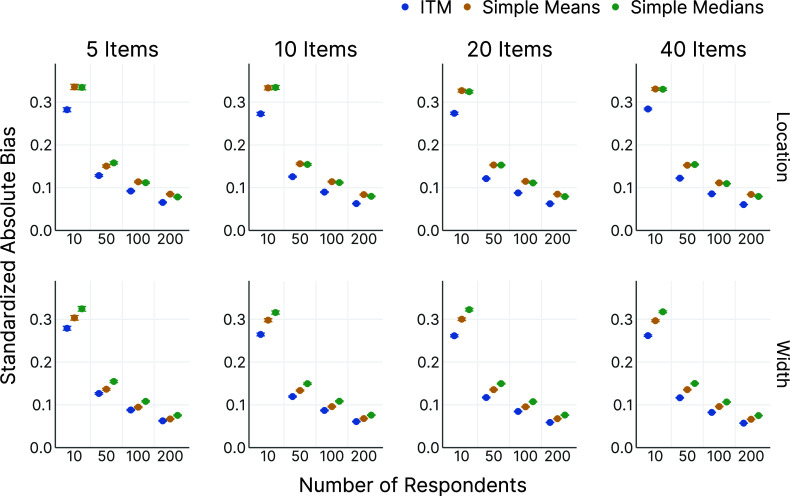
Absolute bias of consensus interval location and width. *Note*: This figure shows the standardized absolute bias (*y*-axis) of the consensus interval location (upper row) and width (lower row) for different numbers of items (columns) and respondents (*x*-axis). The standardized absolute bias was obtained by dividing the condition-wise absolute bias by the true standard deviation of the location or width. Error bars indicate 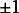 MCSE. Some MCSEs are so small that the upper and lower error bars are indiscernible.

We visualized the absolute bias of the latent interval location and width in Figure 4. The true consensus locations had a higher standard deviation ( 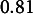 ) compared to the true consensus widths ( 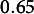 ). Therefore, we divided the absolute bias by the true standard deviations of the respective parameters for ease of interpretation in the figure. The unstandardized performance measures are available in the OSF repository. In all simulation conditions, the ICM has a lower absolute bias averaged over location and width parameters than the simple means and medians. As expected, there is a notable effect of the number of respondents, with a considerably lower bias for higher sample sizes. Increasing the number of respondents from 10 to 50 roughly corresponds to halving the absolute bias for all conditions. The size of the performance difference between the ICM and the simple means and medians remains fairly similar for sample sizes from 50 to 200. Interestingly, the medians performed better than the means for the location dimension, but not for the width dimension. A larger number of items slightly improves the performance of the ICM regarding the recovery of consensus intervals, but this effect is weaker than the effect of the number of respondents. The standardized absolute bias is very similar for the location and width dimensions, which means that both dimensions can be estimated similarly well. We chose to plot both dimensions separately here to illustrate this point. The combined absolute bias, which we defined above in Equation (15), shows a virtually identical pattern of results. Even in the condition with the lowest number of items and respondents (5 items, 10 respondents), the smallest correlation between the estimated consensus locations and the true parameter values is still 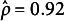 ( 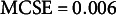 ). This estimate is the same for the consensus widths. In conclusion, the model may be used for the aggregation of interval judgments, even in small samples.

In the OSF repository, we present additional simulation results for all model parameters. These show that the MSE follows a qualitatively very similar pattern to the results of the absolute bias. For all conditions, the ICM had a better performance concerning the MSE than the simple means and medians. Further, in simulation repetitions with a higher bias of the location, the bias of the width tended to be higher as well. Thus, we did not observe evidence for compensatory behavior, where an accurate estimation of one dimension would be associated with a poorer estimation of the respective other dimension.

**Figure 5:**
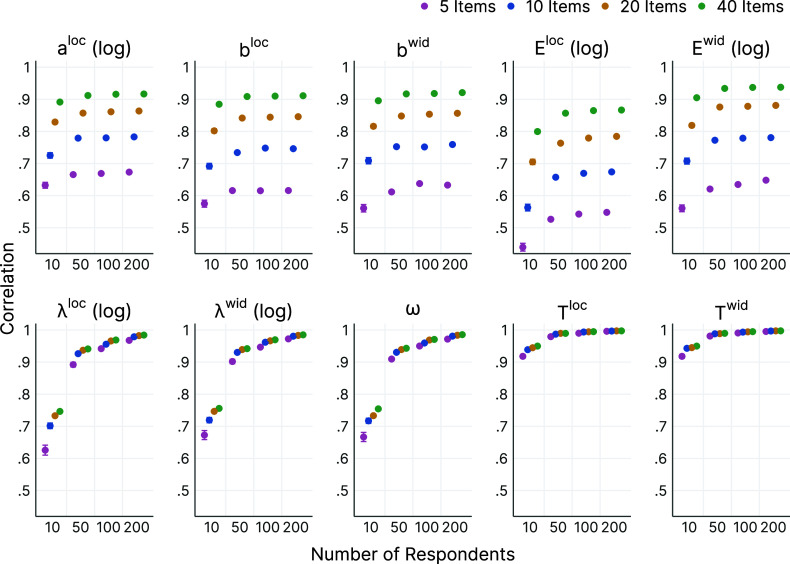
Correlation between true and estimated parameters. *Note*: This figure shows the correlations (*y*-axis) between the true, data-generating parameters and the corresponding model estimates for all parameters (rows) for different numbers of items (columns) and respondents (*x*-axis). Error bars indicate 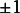 MCSE. Some MCSEs are so small that the upper and lower error bars are indiscernible.

#### 3.5.3 Recovery of other model parameters

Although the main focus of the model is estimating the consensus intervals, the person and/or item parameters may also be of interest in some cases. We visualized the correlation between the true, data-generating parameters and the corresponding model estimates for all parameters in Figure 4. If the focus is on the proficiency parameters for the respondents, it is advisable to collect data for more than 20 items. The correlation between estimated and true parameters for the location proficiency, for example, was 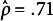 ( 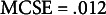 ) with 10 respondents and 20 items, and 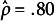 ( 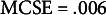 ) with 10 respondents and 40 items. Recovery of the other parameters was generally better than for respondents’ location proficiency, so 20 items should provide a useful lower bound in such cases. If higher reliability is needed, 40 items would be more appropriate.

On the other hand, researchers who are primarily interested in the item parameters can achieve good recovery with 50 respondents, even when using only 5 items. For example, the correlation between estimates and true parameters for the location discernibility was 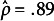 ( 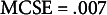 ). Recovery might still be acceptable with less than 50 respondents. However, the next smallest condition in our simulation had 10 respondents, where a correlation of 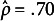 ( 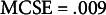 ) was achieved with 10 items. If the recovery performance for such small sample sizes is of interest, the analysis scripts available in the OSF repository allow readers to adapt and re-run our simulation study for other scenarios.

#### 3.5.4 Summary

The results of our simulation study indicate that the ICM performed better than simple means and medians in all conditions we studied. The absolute difference between both approaches became smaller with a larger number of respondents. The number of items did not have a strong influence on the results regarding the consensus intervals. This is not surprising because our performance measures are aggregated across the item parameters. However, the small increases in performance can be explained by the increased precision of person parameter estimates in conditions with larger numbers of items, which in turn helps to estimate the item parameters more precisely. As we standardized the absolute bias, the results can be interpreted as fractions of the true standard deviation, indicating a satisfactory performance of the ICM.

## 4 Empirical example: Verbal quantifiers

To demonstrate the application of the ICM, we reanalyze judgments on verbal quantifiers collected by Kloft & Heck (2024). We use the already cleaned data (accessible from https://osf.io/7azbr). The sample consists of 209 respondents (female: 145, male: 62, and diverse: 2), mainly psychology students, with a mean age of 25.5 years (SD = 8.5).

Participants provided judgments for 16 verbal quantifiers such as “seldom” or “often” using the DRS response format (see Figure 1). For each verbal quantifier, respondents had to assign an interval of probabilities ranging from 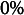 to 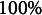 according to the probability that an event described in this way would occur. The full analysis is available in the OSF repository.

### 4.1 Model modification and estimation

Not all parameters described in Section 2 yielded useful estimates in an initial fit of the full model. Specifically, the estimates for the respondents’ response bias parameters 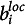 (i.e., systematic shifts in the location dimension) did not differ meaningfully between individuals, as indicated by a variance close to zero. We therefore simplified the model by excluding these parameters.

We estimated the model using the same software as in the simulation study but on a Windows machine. Information on the computational environment is provided in the session info at the end of the analysis script, rendered as an HTML report in the OSF repository. For Bayesian inference, we used the priors described in Section 2.2. We ran four chains of Stan’s Hamiltonian Monte Carlo sampler (Betancourt, 2018) with 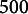 warm-up samples, which were not used for analyses, and 1,000 samples for the computation of parameter estimates. This yielded 4,000 samples per parameter. The adapt_delta parameter was set to 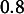 and the range of the initial values of the sampling algorithm for the unbounded parameters was set to 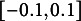 . Convergence was assessed via the 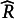 statistic (Vehtari et al., 2021), which was below 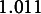 for all parameters. We provide posterior predictive checks in the OSF repository.

### 4.2 Model results

**Figure 6:**
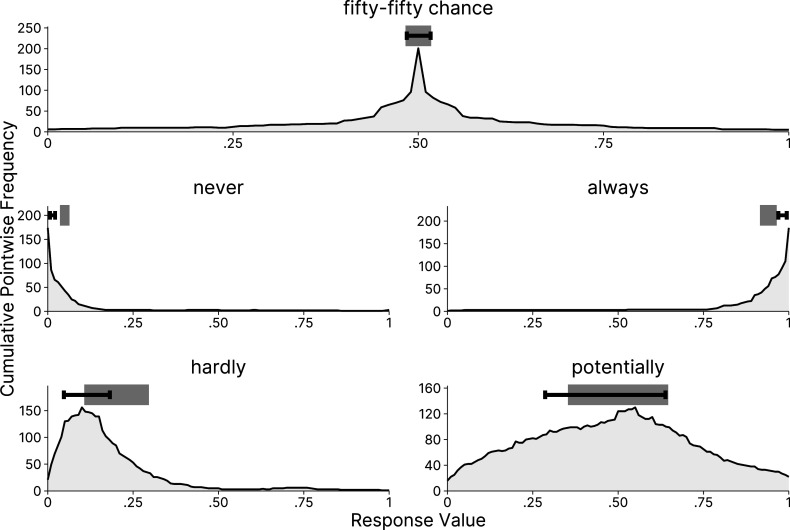
Estimated consensus intervals for verbal quantifiers. *Note*: Black horizontal interval: Consensus interval estimated by the interval consensus model. Gray horizontal bar: Typical interval based on the median location and median width of the observed, logit-transformed response intervals.

Figure 6 presents five examples of estimated consensus intervals (black horizontal intervals) that each resemble the cultural consensus of the sampled respondents, jointly with a simple median of logit-transformed interval responses (gray horizontal bars) and pointwise cumulative frequencies of the empirical interval responses (black density lines).

The “fifty-fifty chance” item (top) was one of the control items in the study, for which respondents were expected to answer with narrow intervals placed in the center of the response scale. The estimated consensus interval is centered on the correct reference value of 50% and very narrow, reflecting the high precision of the verbal statement “fifty-fifty chance.” A substantial proportion of response intervals are wider, as indicated by the density. However, the consensus is still that a “fifty-fifty chance” is a probability very close to 50%. Also, the simple median interval in this case gives a similar estimate of the consensus. The two other control items were “never” (middle left) and “always” (middle right). Figure 6 shows that their consensus intervals were close to the extreme ends of the response scale, as expected for these words. In contrast, the intervals based on the medians are strongly influenced by a skewness of response intervals toward the center of the scale. Overall, the three control items demonstrate that the ICM provided more meaningful estimates of the interval consensus than simple aggregation via the median.

The item “potentially” (bottom right) provides an example of a typical pattern found for most of the verbal quantifiers. The simple median interval was more strongly influenced by the concavely shaped longer tail of the distribution of response intervals, while the consensus interval estimated by the model was more representative of the convexly skewed shorter tail. This trend also appeared for the item “hardly” (bottom left). The model-based estimate of the consensus interval was more representative of the empirical distribution, while the simple median interval was shifted toward the center of the scale, demonstrating a stronger influence of the inwardly skewed outliers of the empirical responses. Overall, the model estimates provided a better representation of where the bulk of response intervals were located.

**Figure 7:**
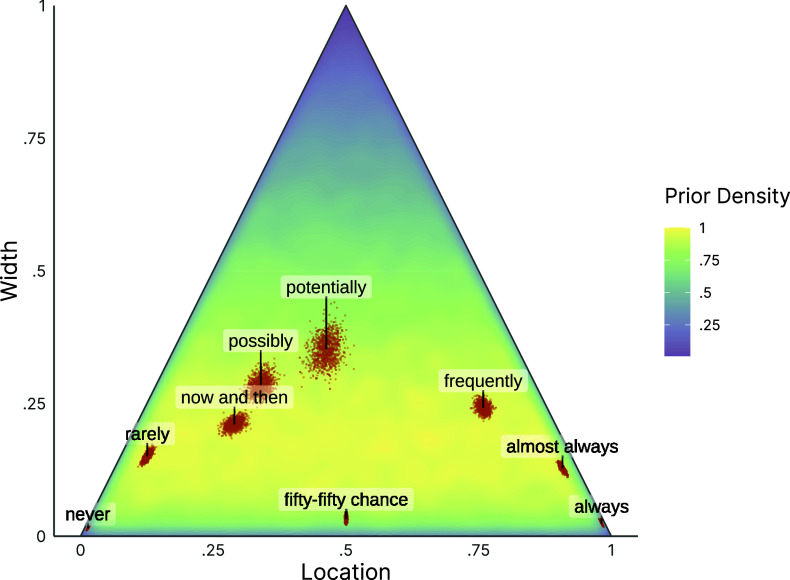
Prior and posterior distributions for the cultural consensus intervals. *Note*: Orange points: 1,000 posterior draws for each verbal quantifier. Purple to yellow density in the background: prior density estimated from 1,000,000 samples and standardized to a maximum density of 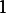 . The prior on the marginal distribution of interval widths is 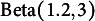 . The prior on the marginal distribution of interval locations, conditional on the interval width, is 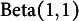 .

Figure 7 displays posterior draws of the consensus intervals for a selection of verbal quantifier items, jointly with the prior density of the model (a plot of all verbal quantifiers can be found in the OSF repository). The plot includes the three control items “never,” “fifty-fifty chance,” and “always” at the bottom, reflecting a shared consensus that the meaning of these quantifiers in terms of probabilities is clear (i.e., the width on the *y*-axis is estimated to be very small). The other quantifiers have larger widths, indicating a consensus that using these words comes with more ambiguity. While the posterior distributions for most control items are relatively peaked and precise, more vague quantifiers, such as “potentially,” also show higher estimation uncertainty. The model allows us to distinguish two types of uncertainty: First, the increased width, as shown by large values on the *y*-axis, of the item “potentially” indicates a latent consensus that the item has a wider range of plausible meanings. Second, the wider posterior distribution, as shown by the distribution of posterior samples, indicates that the estimation certainty for this inference is lower than that for the remaining items. Furthermore, the prior density in the background of Figure 7 also illustrates that our weakly informative prior was an appropriate choice for this application, as most posterior distributions are located in areas of relatively high prior probability.

The estimated correlation of respondents’ proficiencies for the location and the width dimension (see also Table 1, column 1) was 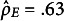 ( 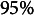 HDI 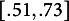 ). Substantively, this means that respondents who answered highly consistently with respect to interval locations were also highly consistent regarding interval widths, that is, when judging the variability in how the quantifiers are being used.

**Figure 8:**
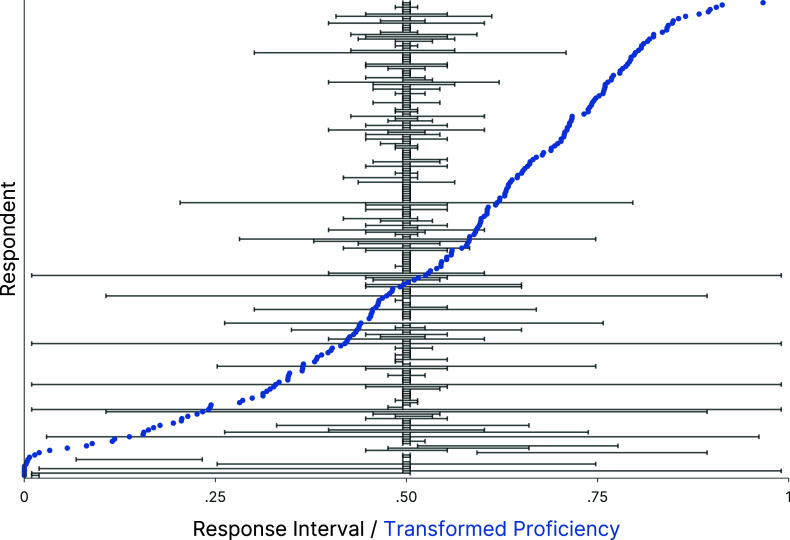
Empirical interval responses and estimated proficiencies for the item “fifty-fifty chance.” *Note*: Black horizontal bars: Empirical response intervals. Blue dots: Estimated proficiencies, computed per person as the mean of the standardized posterior medians for the location and the width proficiency, transformed to normal quantiles.

Figure 8 provides insights into how the estimated proficiencies relate to empirical interval responses. The empirical responses of all participants to the verbal quantifier “fifty-fifty chance” (black intervals) are shown jointly with the corresponding individual proficiency estimates (blue points). For illustration, the two-dimensional proficiency estimates are collapsed within each individual by taking the mean of the location and the width parameter. Respondents are ordered by their proficiencies from high (top) to low (bottom). The respondents with the highest proficiencies (upper half of the *y*-axis) mostly provided relatively narrow intervals located at the center of the response scale. In the lower half of the *y*-axis, respondents provided much wider response intervals, some of which were necessarily located at the center of the scale due to their large width. Those respondents with the lowest proficiency at the bottom of the *y*-axis mostly failed to place the interval at the center of the response scale. This shows that the proficiency estimates may be useful for diagnosing non-effortful responding. The model also enables us to automatically downweight the responses of unreliable respondents. This is achieved by the person proficiency parameters, which assign higher error variances to respondents providing inconsistent response patterns (see Section 2.1). Consequently, we do not have to exclude respondents from the data based on possibly arbitrary filtering criteria.

Regarding item parameters, the discernibilities of consensus locations and widths (see also Table 1, column 2) were correlated negatively with 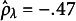 ( 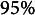 HDI 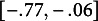 ). This correlation should be considered with caution since it is driven by the control items (“never,” “always,” and “fifty-fifty chance”). These had especially high location discernibility estimates, above the mean, and especially low width discernibility estimates. At the same time, all other items’ location discernibility estimates were below the mean, and their width discernibility estimates were above the mean. We initially selected these verbal quantifiers as control items because they have a clear implication regarding the probability assigned to them, that is, “never” 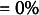 , “always” 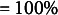 , and “fifty-fifty chance” 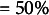 . The high location discernibility indicates that respondents overall interpreted these quantifiers in the assumed way.

To check whether the negative correlation between location and width discernibilities was just due to the control items’ influence, we re-fitted the model, excluding the three control items. As expected, the correlation was no longer negative and even changed to a large positive value with 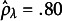 ( 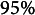 HDI 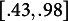 ). This means that items with an easy-to-detect consensus location also tended to have a consensus width that was easier to detect. At the same time, the correlation for respondents’ location and width proficiencies was reduced to 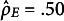 ( 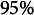 HDI 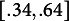 ). Since respondents who participated seriously were likely able to set a reasonably accurate location and width for the control items, these items might have artificially inflated the correlation between person proficiencies. Therefore, the lower correlation provides a more conservative, and arguably more appropriate, estimate. In conclusion, our empirical example shows that item parameters, such as discernibility, can facilitate manipulation checks or may be used to exclude poorly performing items.

## 5 Discussion

We proposed the ICM as a means of estimating the shared consensus of a group of individuals regarding continuous, bounded intervals. In a simulation study, the consensus model outperformed the descriptive approach of simply averaging the response intervals or taking the median. We also showed that the model can be estimated with as little data as five items and ten respondents. We further illustrated the application of the proposed model to empirical data in the case of interval judgments for verbal quantifiers, such as “rarely” or “frequently.” The model-based analysis led to valid conclusions for control items such as “always” and allowed us to detect and downweight the responses of unreliable respondents.

The results of a preliminary simulation study showed that our choice of the ILR transformation over the amalgamation log-ratio transformation was justified, as the former was more robust to model misspecification (i.e., when using a different transformation for model fitting than for data generation). The ICM showed good convergence and a better performance in terms of absolute bias and MSE than aggregation via simple means and medians. Even in the “hardest” conditions with small numbers of respondents and items, divergent transitions occurred only in a small proportion of models. If such divergences occur in empirical research, one may need to specify more informative prior distributions. The possibility of defining prior distributions is a strength of the Bayesian approach, which allows for incorporating expectations and knowledge about the consensus intervals, thereby facilitating robust estimation even in small datasets (Krypotos et al., 2017). However, not all parameters may be necessary, nor can they always be estimated with sufficient precision in every use case. Issues may emerge due to low variance between respondents, as in our empirical example, or due to noisy data. Under such circumstances, auxiliary parameters for person biases may be removed as needed. Moreover, even if the proficiency parameters cannot be estimated precisely, the model reduces to estimating an unweighted mean consensus interval for each item.

In our empirical example, estimated consensus intervals were centered on the factually true value in the case of “fifty-fifty chance,” and on the mode of the distribution in the case of the item “hardly.” Compared to computing simple means or medians, the model-estimated consensuses appeared to be more robust against extreme responses from individuals with low proficiency. The model can therefore be used to obtain higher-quality estimates of a latent consensus interval. While simple trim-and-average heuristics (Gaba et al., 2017; Lyon et al., 2015; Park & Budescu, 2015) could be useful in this regard, our model-based approach offers the advantage of providing estimates for the proficiency of respondents and the discernibility of items. These estimates may be used for diagnostic purposes, as illustrated by the analysis of control items in the empirical example. Further, the ICM could be extended to an explanatory model, for example, by incorporating latent regressions for the item or person parameters (for an example of how this might be implemented, see Heck et al., 2018). This might be relevant for researchers investigating potential predictors of the respondents’ proficiency or the discernibility of items.

We confined ourselves to studying a specific version of a consensus model for interval responses, but there are several possibilities for extensions of this model, which we did not cover in the present article. We chose the ILR as the link function for our model and investigated one alternative link function (Smithson & Broomell, 2024). However, there might be other appropriate link functions that we are not aware of. Further research might focus on developing alternative link functions to find an optimal match between different link functions and certain types of applications of the interval response format (Ellerby et al., 2022; Kloft & Heck, 2024), or explore model versions that do not require a link function at all (e.g., by relying on the Dirichlet distribution; Kloft et al., 2023).

The model assumes a single, shared consensus interval for each item. However, in some applications, it is plausible to assume more than one latent consensus for different unknown groups, in other words, latent classes of respondents (see Anders et al., 2014). We also did not cover the case where the latent consensus is a single point, for example, a specific risk probability, while responses are collected with an interval format, for example, by judging the range of plausible probabilities, as in forecasting (Gaba et al., 2017; Peeters & Wolk, 2017). This case warrants the development of a new model which estimates this point consensus based on interval responses. Such a model requires further assumptions about where the best guess of a respondent is located within an observed response interval. Alternatively, one may fit the ICM proposed in the present work to derive a consensus interval for the point truth and then judge forecasting performance based on the coverage of the target value. However, the model will not provide a single best guess within the estimated consensus interval. The estimated consensus intervals differ conceptually from confidence intervals for point estimates in a classical consensus model because they reflect the subjective (meta-)uncertainty *within* respondents rather than the estimation uncertainty *between* respondents.

Regardless of the specific model being used, when researchers are interested in a latent underlying point quantity but still ask participants to provide interval responses, it is important to provide clear instructions about the meaning of the interval bounds. For example, the interval bounds could represent the lower and upper bounds of plausible values or, alternatively, a symmetric interval of uncertainty around the best guess (Kloft & Heck, 2024). A simpler alternative solution might be to provide participants with three response values: one for their best guess, and two for the lower and upper bounds of the interval. In this case, the ICM and the ILR function can be extended to a third dimension, representing the asymmetry of the best guess relative to the encompassing interval bounds. The development of such extended models is beyond the scope of our article and provides promising avenues for future research.

## Data Availability

All data and analysis scripts as well as the preregistration of the simulation study are available at the Open Science Framework (OSF): https://osf.io/r32by/. The README file of this repository (see the “Github” tab on the “Files” page), which is also mirrored in the repository’s Wiki home page, contains a table of contents describing where specific materials mentioned in the article are located. We have also implemented the methods used in this article in an R package called *intervalpsych*, which is available on CRAN: https://doi.org/10.32614/CRAN.package.intervalpsych (for the development version, see https://github.com/matthiaskloft/intervalpsych).

## Abbreviations and parameter interpretations

- CCT: Cultural consensus theory
- ICM: Interval consensus model
- DRS: Dual range slider
- ILR: Isometric log-ratio function
- MCMC/HMC: Markov Chain Monte Carlo/Hamiltonian Monte Carlo
- HDI: Highest density interval (for a given posterior distribution; Bayesian)
- : Statistic for the diagnosis of MCMC convergence
- MCSE: Monte Carlo standard error
- MSE: Mean squared error
- OSF: Open science framework
- ADEMP: Aims data-generating mechanisms estimands methods performance measures
- Data Definitions:
  1. : Lower and upper bounds of interval response
  2. : Interval response in its simplex representation/compositional format
  3. : Interval response in its simplex representation after adding a padding constant to replace zero components
  4. : Logit-transformed interval response on the unbounded scale
- Model Parameters of the ICM:
  1. ,  : Respondent’s latent appraisal of interval location and width
  2. : Person scaling bias for the interval location
  3. ,  : Person shifting bias for the interval location and width
  4. ,  : Person proficiency to detect the consensus interval location and width
  5. ,  : Latent consensus interval location and width on the unbounded scale
  6. : Latent consensus interval lower and upper boundary on the bounded response scale with reversed zero-handling transformation
  7. ,  : Item discernibility for the consensus interval location and width
  8. : Residual correlation between location and width dimension
